# Cavitation bubble dynamics in a vicinity of a thin membrane wetted by different fluids

**DOI:** 10.1038/s41598-021-83004-7

**Published:** 2021-02-10

**Authors:** Žiga Lokar, Rok Petkovšek, Matevž Dular

**Affiliations:** Faculty of Mechanical Engineering, University of Ljubljana, Askerceva 6, 1000 Ljubljana, Slovenia

**Keywords:** Mechanical engineering, Condensed-matter physics, Fluid dynamics

## Abstract

Understanding and controlling the interaction of cavitation bubbles and nearby material is becoming essential optimization of various processes. We examined the interaction of a single bubble with a membrane with different fluids on each side of it. Significant differences in bubble behavior depending on the fluid properties were observed, while the influence of membrane properties was less pronounced. The study has important implications, such as optimization of sonoporation (targeted drug delivery) where the mechanism, by which the permeability of the membrane is increased, is still not well understood. These results show that the focus of the optimization process should, in the first place, lie on the properties of liquids, rather than the mechanical properties of the membrane itself.

## Introduction

Several decades ago, cavitation, due to its aggressive nature, used to be considered solely as one of the most ubiquitous problems in turbomachinery. Yet nowadays, the effects that accompany it, such as strong shear flows^1^, jets^2^, high local temperatures^3^, shock waves^4^, rapid depressurization^5^ and supersonic flow^6^, are being exploited for numerous applications in medicine^7^, chemistry^8^, biology^9^, in environmental protection^10,11^ and in liquid food preparation. One of the most interesting application of cavitation bubbles is surely sonoporation, where ultrasound frequencies are used to excite the bubbles near a cell membrane to increase its permeability and to promote the drug delivery process^12^. Yet, as in many other cavitation applications, the exact mechanism of sonoporation is not yet determined, mainly due to complicated nature of the process^13^. Understanding and controlling the interaction of cavitation bubbles and nearby material is therefore becoming essential.

Studies of cavitation bubble behavior in an anisotropic field (presence of boundary, shear flow, gravity, free surface etc.) are numerous—here the work by Supponen et al.^14^, where the researchers proposed a so-called anisotropy parameter in an attempt of unification of the understanding, stands out. Many studies of bubble interaction with elastic material can be found, but these are primarily limited to a bulk material^15–19^. Studies of bubble interaction with thin membranes, which for example aim to investigate the mechanisms of sonoporation process^20,21^, primarily focus either on the properties of the membrane (trying to make it mimic a biological structure) or the properties of the liquid. However, in existing works the liquid, which wets the membrane, is the same on both sides and no attention has been given to the influence of the fluid properties that wet the membrane from each side.

In the present study we observed interaction of a single cavitation bubble near different types of membranes, which separated liquids with different properties. It is shown that regardless of the stiffness of the membrane the bubble response is primarily a function of the properties of the fluids, which wet the membrane from each side, rather than the properties of the membrane itself. The experiments were performed on a larger scale, with simpler (not biological) materials, but effort was put into preparation and execution of experiments so that we can, at least partially, apply conclusions to, for example, sonoporation processes (bubble dynamics does not change significantly when scaled down, also the ratio between the maximal bubble diameter and the membrane thickness in the present experiments is in the same order of magnitude as one would expect in a sonoporation process).

The observations are opening a new chapter in the understanding of bubble membrane interaction, which, in the past, based solely on the properties of the membrane, and not on the fluids.

## Methodology

Due to the complex nature of the phenomena, we approach the investigation at a larger scale, but still small enough, for the results not to be interfered by severe scale effects.

### Experimental approach

**Figure 1:**
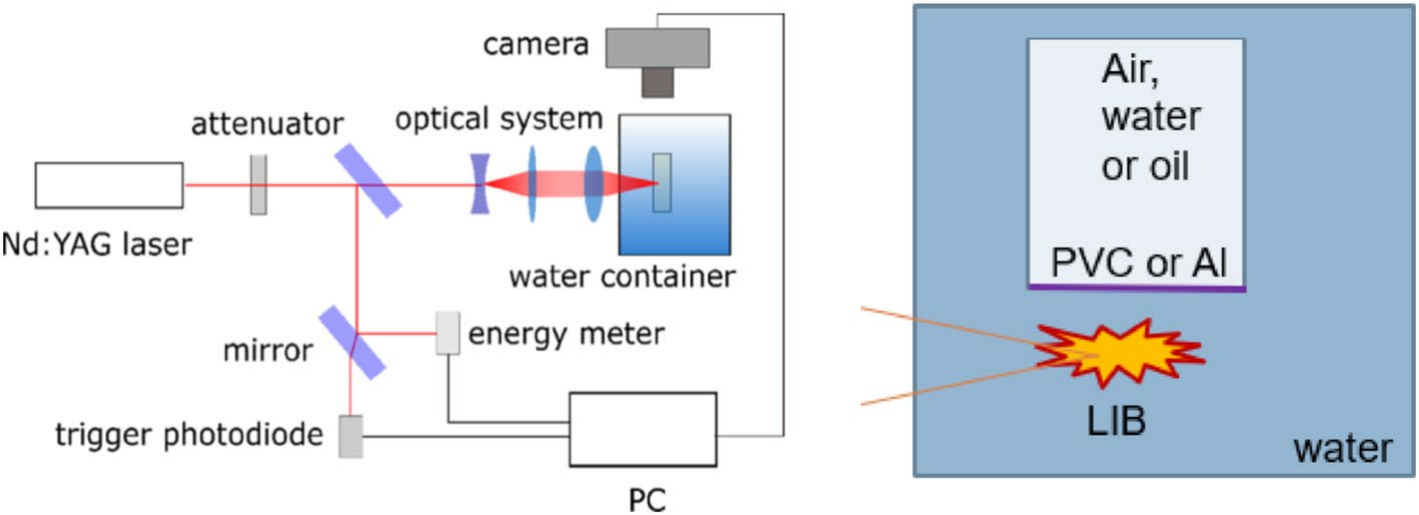
Experimental setup.

Figure 1 shows the experimental setup. A Nd:YAG laser. Beam is expanded and then tightly focused with a lens, NA of the optical system is 0.2 in water. Optical breakdown occurred in the focal area, followed by bubble formation. The conversion efficiency was approximately 20% for an unbounded bubble (calculated as the fraction of potential energy of the maximal achievable bubble size R_b_ = 1.1 mm ($E={p}_{at}{V}_{b}$) and the laser energy delivered to the breakdown spot (already considering the losses in optical system and absorption in water—roughly 45% of the initial laser energy of 6 mJ)). In the presented experiments we used lower laser energy, what resulted in a bubble with R≈0.9 mm. This is comparable to the setups of other researchers^22,23^. No significant differences in efficiency due to the changes in liquids or membranes is expected—all energy is deposited at breakdown, when membrane is far enough to not to be in direct contact with plasma.

The evolution of the cavitation bubble was recorded by a high-speed camera Photron Fastcam SA-Z with a frame rate of 210,000 fps. The whole region of interest was 10 × 4 mm big and resolved by 384 × 160 pixels, leading to the pixel size of approximately 25 μm. LED light source Ryobi One + (50,000 lm) served as a backlight illumination source.

To determine the influence of the membrane properties, two very different materials were selected. Both foils were 10 μm thick. The measured elastic modulus of aluminum one was E_Al_≈35 GPa. the elastic modulus of the plasticized PVC was several orders of magnitude lower—E_PVC_≈50 MPa.

Membrane was fixed to a plastic cuvette filled by air, water or oil. To fix the foil, special attention was given, not to pre-stretch it. A thin layer of glue (cyanoacrylate) was first applied to cuvette sides. A larger sheet of foil was positioned on a flat surface and pressed against the cuvette. The excess of the foil was removed once glue set in place. Distilled water was used both inside the cuvette as well as in the water container to decrease the amount of impurities. Vegetable oil with viscosity 50 cP and density 915 g/cm^3^ was used.

**Figure 2:**
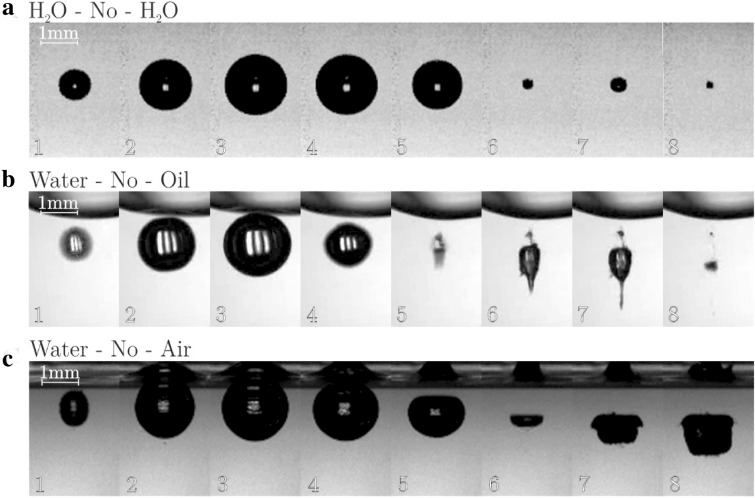
Typical bubble evolution for (**a**) water-water (unbounded bubble), (**b**) water–oil, (**c**) water–air interface at γ  = 0.7.

Cuvette with membrane was oriented with membrane down and fixed to a 3-D positioning stage to allow precise manipulation of its position. As shown in Fig. 2, laser is incident parallel to the membrane.

## Results

Several exemplary cases are shown first. These all correspond to same dimensionless ratio of bubble-interface distance h and maximum bubble radius R_max_: $\gamma =\frac{h}{{R}_{max}}=0.7$ (except the case of a bubble in an “infinity”, top sequence in Fig. 2). The time and size scales are the same in all figures. The first frame that is shown occurs 10 μs after the optical breakdown, the time difference between the frames is always 33 μs.

Figure 2 shows the bubble dynamics for the case where there is no physical boundary placed between the two fluids (for the water-water case, this is of course a situation of an unbounded bubble).

For the case of unbounded bubble (H_2_O–No–H_2_O) the growth and the collapse are of course symmetrical. The size of the rebounded bubble is small compared to the maximum bubble radius. This points to the fact that the majority of the energy, which was released at the instant of collapse transferred into the shock wave emission.

When the plasma discharge is positioned near an oil interface (H_2_O–No–Oil), the growth phase is not significantly influenced—the maximum radius is the same. The collapse is obviously asymmetrical, a jet pointing away from the interface forms. Splitting of the bubble at the rebound is witnessed—a very small bubble moves toward the interface, while a much larger one follows the jet.

For the case of bubble evolution near a free surface (H_2_O–No–Air) the most obvious feature is a very large bubble rebound. The jet, which is penetrating the bubble has a large diameter (almost the size of the rebounded bubble) and is very slow—most of the energy is transferred to the building of the liquid cone on the free surface.

**Figure 3:**
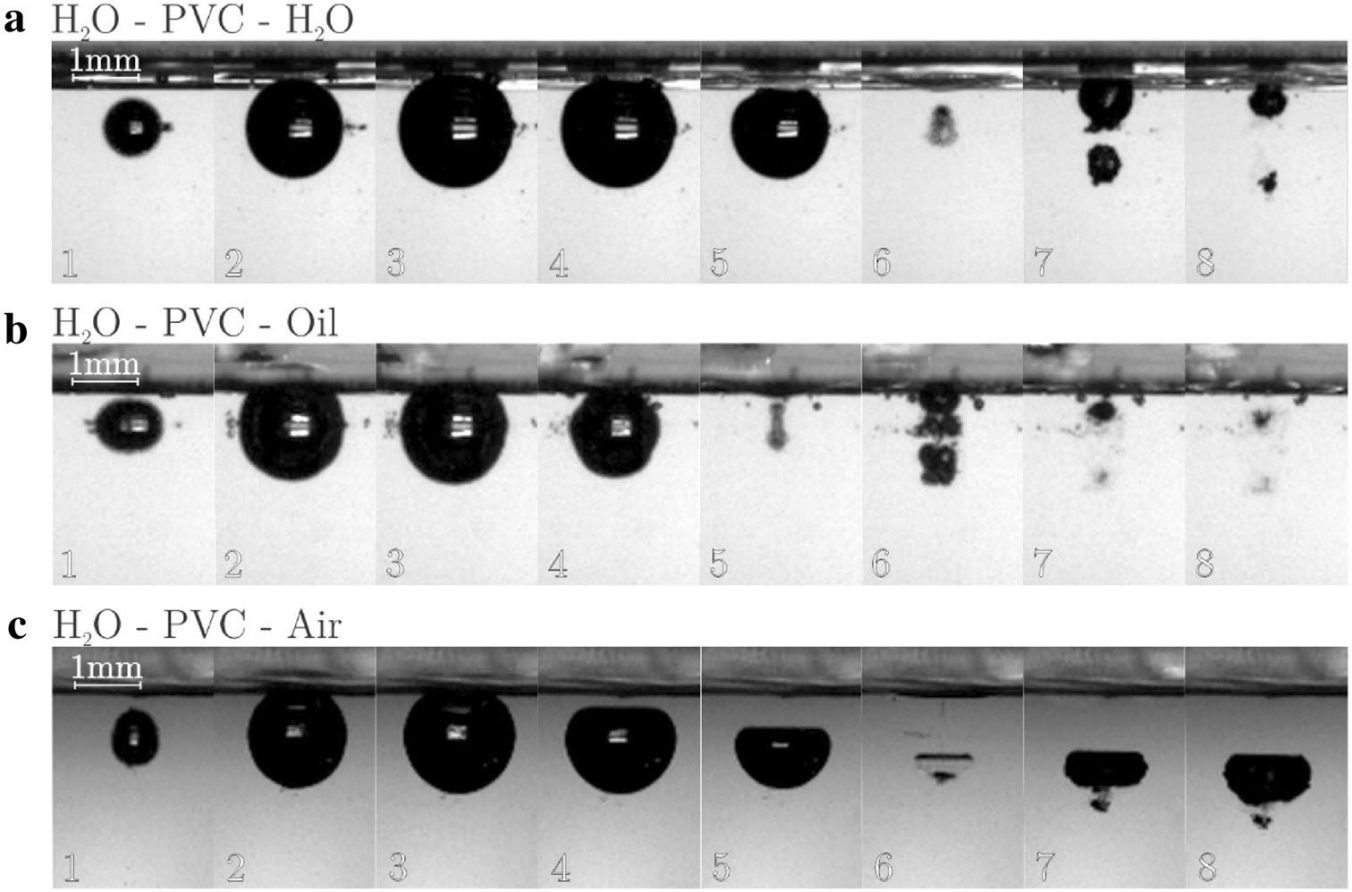
Typical bubble evolution for (**a**) water-PVC-water, (**b**) water-PVC-oil, (**c**) water-PVC-air interface at γ  = 0.7.

In Fig. 3 a 10 μm PVC membrane is separating the fluids.

Placing a thin membrane to separate the fluids completely changes the bubble evolution. For the case of H_2_O–PVC–H_2_O, the rebound shows separation of initial volume into two bubbles of comparable size. The rebounded volume is also much larger than in the case of unbounded bubble (Fig. 3, top).

In the case of H_2_O–PVC–Oil similar dynamics is observed—at rebound the bubble splits into two equal parts. Unlike in the case of no membrane where jet is directed away from the oil, here significant portion of energy is directed towards the membrane. Jet resulting from breakdown is occasionally even sufficient to penetrate the membrane.

Interestingly, the H_2_O–PVC–Aircase is very similar to the case, where no membrane separated the fluids (Fig. 3, bottom). This is a result of very low inertia, which is offered by air (and the PVC membrane).

One general observation in cases H_2_O–PVC–H_2_O and H_2_O–PVC–Oil is that the two separated bubbles, which form at the rebound have different sizes—the one closer to the boundary is always larger.

**Figure 4:**
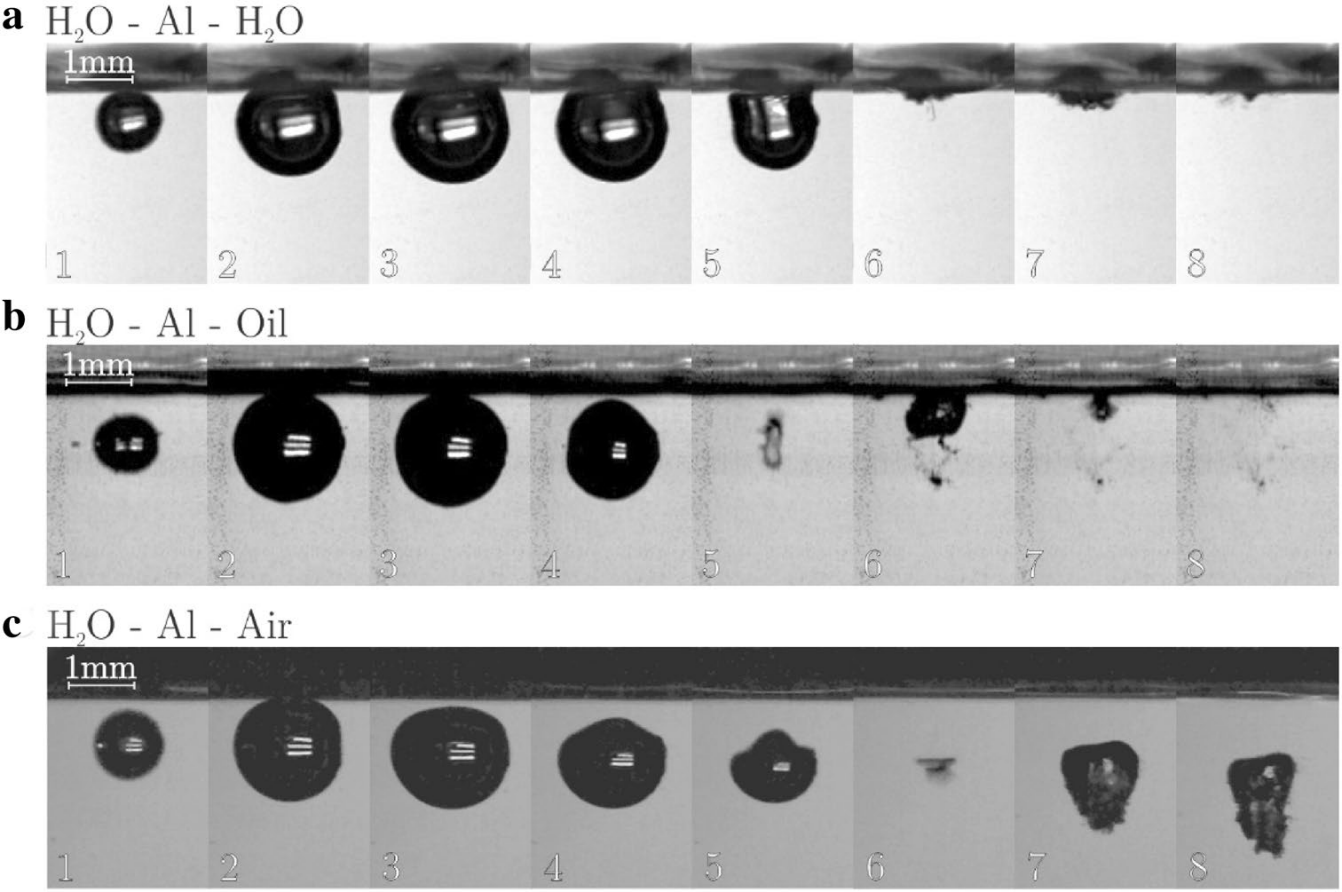
Typical bubble evolution for (**a**) water–Al–water, (**b**) water–Al–oil, (**c**) water–Al–air interface at γ  = 0.7.

Finally examples of case at γ = 0.7 with a 10 μm aluminum membrane separating the fluids is shown in Fig. 4.

Aluminum membrane is stiffer than the PVC one. Hence, one would expect significant influence on the bubble evolution. However, this is not really the case. In the case of H_2_O–Al–Air, the stiffer aluminum membrane still does not change the volume or the jet formation—although the rebounded bubble is somewhat more elongated than in the case of PVC or no membrane, also it exhibits a “mushroom shape” during primary bubble collapse. On the other hand, H_2_O–Al–H_2_O and H_2_O–Al–Oli cases show more pronounced difference between the rebound bubble sizes—the bubble closer to the boundary is much larger than the one further away.

Comparing cases “a” (H_2_O–membrane–H_2_O), cases “b” (H_2_O–membrane–Oil) and cases “c” (H_2_O–membrane–Air) in Figs. 2, 3, 4 we find no influence of the interface or membrane on the collapse time—only the boundaring fluid had some influence—the time dynamics is virtually the same. Likely the membranes are flexible enough to minimize the effect of prolongation of the collapse time near a boundary, which was reported by Vogel and Lauterborn^22^.

Influence of fluids and membranes was explored for different γ. Two parameters can be assessed simply and accurately: the cumulative volume of the rebound bubble(s) and their displacement. The volume was calculated by firstly measuring the area the bubble occupies and then assuming the rotational symmetry along the axis through the centre of the bubble in the direction perpendicular to the foil/interface. The displacement is determined by measuring the distance between the initial centre of gravity of the bubble and the one at the rebound (at the moment of maximum rebounded size of the bubble).

**Figure 5:**
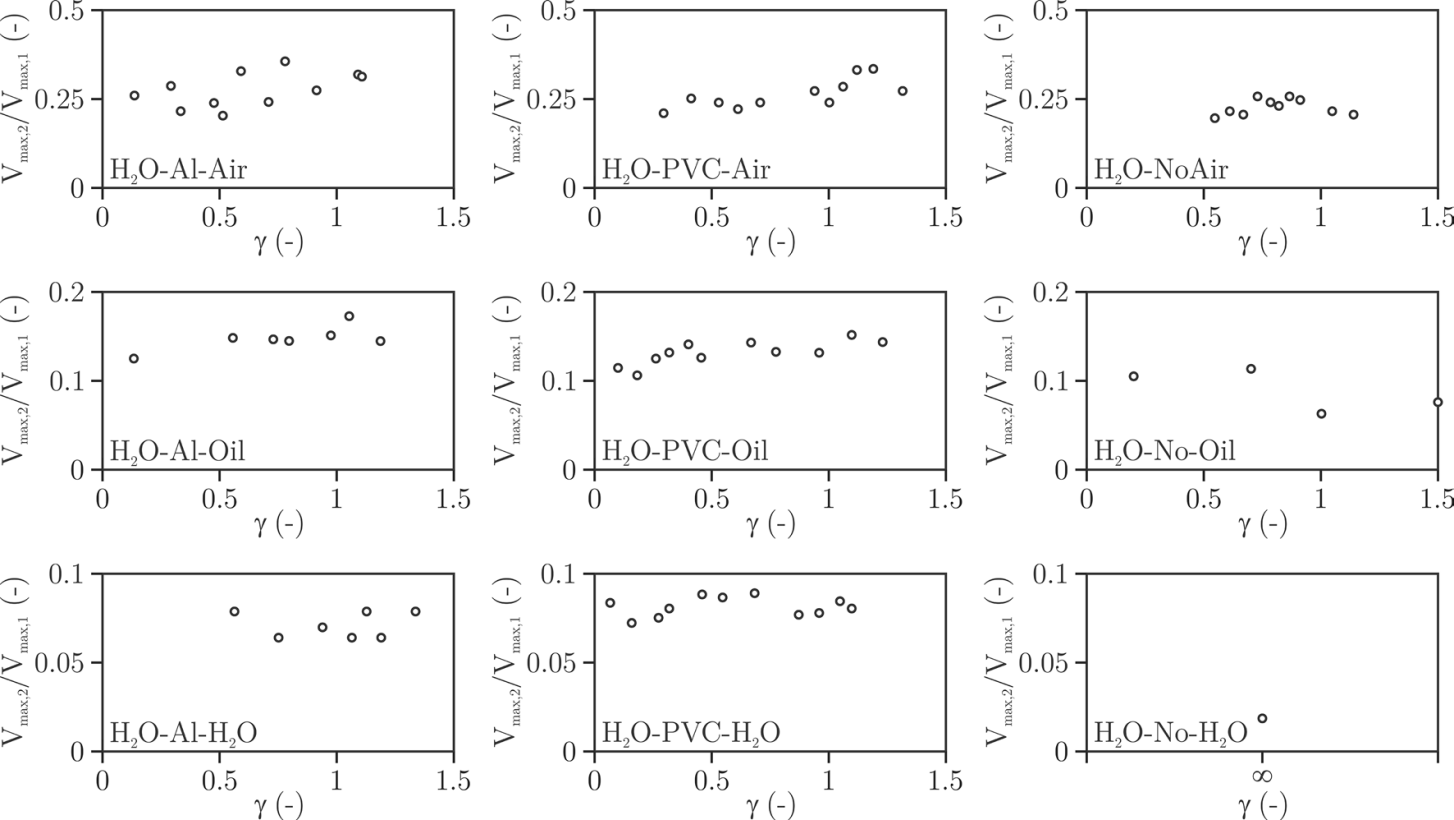
Cumulative volume of the rebounded bubble(s) nondimensionalized against the maximum volume of the bubble as a function of γ and the type of fluid-interface-fluid arrangement. Note different y scale for different fluids.

Figure 5 shows ratio of cumulative volume of the rebounded bubble(s) nondimensionalised against the maximum volume of the bubble for different γ values.

The first obvious observation is that there is no significant influence of the nondimensional distance of the bubble form the interface. This is expected in the investigated range of γ, where the bubble collapse is severely asymmetrical. At larger distances, for example in the case of H_2_O–No–Oil at γ  = 1.5, the bubble collapse deviates less from its initial spherical shape, hence its rebound is smaller. This can, of course, be extrapolated to the ideal, unbounded case (H_2_O–No–H_2_O).

The volume of the rebound is influenced only by the neighboring fluid. The type of membrane is not important at all, despite significant difference in their stiffness. The volume of the rebound decreases from approximately 30% for the case of air to about 7% for the case of water. In an ideal case (H_2_O–No–H_2_O) the ratio of volumes is only 2%. The closer the densities of neighboring fluids lie, the larger is ratio of the energy which is transferred to the shock wave and the smaller is the bubble rebound. Another effect could lie in the difference in the fluids viscosity, however in our preliminary study ^24^ on bubble dynamics near a water–oil interface we found that the differences in the behaviours of the bubble dynamics is insignificant, when oils with similar density and different viscosity are used.

**Figure 6:**
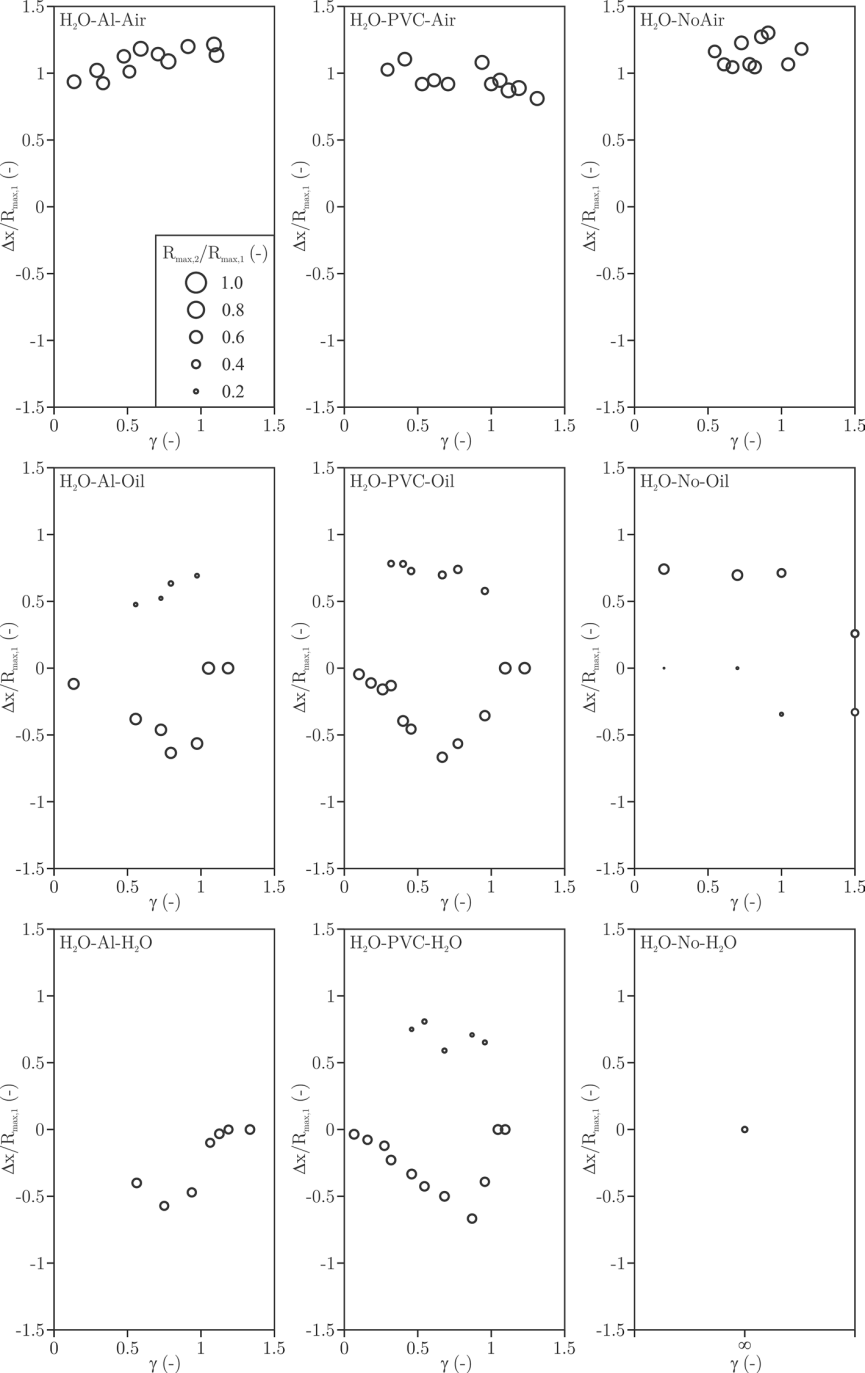
Displacement of the rebounded bubble(s) together with their radius (given by the size of the circle) nondimensionalized against the maximum radius of the bubble as function of γ and the type of fluid-interface-fluid arrangement.

Figure 6 shows displacement of the rebounded bubble(s) together with their radius (given by the size of the circle) nondimensionalized against the maximum radius of the bubble. Since the bubble is not perfectly symmetrical, the radius is considered as one that would form a sphere of the same volume as the real volume of the bubble. Distance at maximum rebound is used. Positive values of displacement mark movement away from the interface and negative values towards the interface.

Air, due to its negligible inertia, offers little resistance, hence the bubble is not stretched and does not split, regardless of the presence of the membrane. Also, the radius of the rebounded bubble is in the range of 60–70% of the maximal radius (volume ratio of approximately 30%, Fig. 5).

If either oil or water is wetting the membrane, in a certain range of γ the bubble splits, regardless of the membrane type. Three regimes can be observed. After the collapse the rebounded bubble remains attached to the membrane at approximately γ  < 0.5, in a range of 0.5 <  γ  < 1 the bubble splits into two rebounded bubbles and when γ  > 1 the bubble does not move or split at the rebound.

The rebounded bubble, which moves towards the interface is larger if membrane is present. This may be due to the wettability of the membranes used in the present experiment—both are hydrophilic and attract the bubble. Only in the case of H_2_O–No–Oil the bubble closer to the membrane is much smaller than the one displaced away from the interface.

One, not so obvious difference is that the density of the neighboring fluid influences the size of the rebounded bubbles. In the case of air, we cannot observe bubble splitting—all of the energy that went into rebound manifests in the bubble, which moves away from the interface. For the case of oil the energy is shared—more of it goes into the bubble, which is closer to the interface, but the opposing bubble (which moved away from the interface) still has significant size. The trend is continued for the case of water, where only very small bubble (even no bubble for the case of Al membrane) can be observed to move away from the interface, while a larger one remains in contact with the membrane.

Interestingly, the results presented here also fit well with the past study by Orthaber et al.^25^, where bubble collapse near a membrane wetted by water from both sides was observed. The elastic modulus of the polyethylene membrane was 0.24 GPa—an order of magnitude stiffer than the PVC one and an order of magnitude more elastic than the Al one used in the present experiment. An in-depth analysis was not performed in the work by Orthaber et al.^25^, but existing results are comparable to the case H_2_O–Al–H_2_O and H_2_O–PVC–H_2_O cases of the present work. This again proves that the membrane properties play lesser role in the dynamics of bubbles.

## Conclusions

The present study opens a new look into the interaction between a bubble and a membrane. It shows that the stiffness of the membrane has a small influence on the bubble dynamics. The topology of the bubble collapse is predominantly dependent on the properties (density) of the fluid that wets the opposite side of the membrane. Also, the density of the fluid that wets the opposite side of the membrane determines the portion of the energy which is transferred into the shock wave emission and the portion that is transferred into the rebound.

This understanding is essential, for example, for optimization of sonoporation process, where one wants to create aggressive bubbles, which collapse in the vicinity of the membrane, transfer the majority of the energy into shockwaves or to a jet oriented towards the membrane in order to make it more permeable for drug delivery.

One of the possible paths for further exploration is scaling down the present experiment. This is essential for developing more exact methodologies for optimisation of sonication technologies, where the bubbles are several orders of magnitude smaller than the ones used in the present study.
